# Bioactivities and molecular networking-based elucidation of metabolites of potent actinobacterial strains isolated from the Unkeshwar geothermal springs in India†

**DOI:** 10.1039/c8ra09449g

**Published:** 2019-03-28

**Authors:** Gajanan T. Mehetre, Vinodh J. S., Bhushan B. Burkul, D. Desai, Santhakumari B, Mahesh S. Dharne, Syed G. Dastager

**Affiliations:** NCIM Resource Centre, CSIR-National Chemical Laboratory Pune India sg.dastager@ncl.res.in; Academy of Scientific and Innovative Research (AcSIR) Ghaziabad India; Centre for Materials Characterization, CSIR-National Chemical Laboratory Pune India; National Center for Nanoscience and Nanotechnology, University of Mumbai India

## Abstract

The bioactive potential of Actinobacteria endemic to hot springs has rarely been investigated. This study highlights the cultivable diversity and bioactivities of Actinobacteria associated with the Unkeshwar hot springs, India. Potent strains were evaluated for their biosynthetic potentials and metabolite analysis was performed using effective dereplication molecular networking tools. A total of 86 actinobacterial strains were isolated and grouped into 21 distinct genera, based on 16S rRNA gene sequence analysis. These strains included rare members such as *Micromonospora*, *Marmoricola*, *Actinomadura*, *Cellulomonas*, *Cellulosimicrobium*, *Janibacter*, *Rothia*, *Barrentisimonas*, *Dietzia* and *Glycomyces*. In antimicrobial screening, *Micromonospora* sp. strain GH99 and *Streptomyces* sp. strain GH176 were found to be potent antimicrobial strains. The metabolic extracts of these strains exhibited strong antimicrobial activity against *Staphylococcus epidermidis* (NCIM 2493), *Shigella flexneri* (NCIM 5265), *Klebsiella pneumonia* (NCIM 2098), and *Salmonella abony* (NCIM 2257). The extracts also displayed strong anti-biofilm and anticancer activities against *Pseudomonas aeruginosa* (NCIM 5029), *Acinetobacter junii* (NCIM 5188) and breast cancer cell line MCF7, respectively. Both strains also tested positive for the presence of the PKS biosynthetic gene cluster in their genomes. To effectively delineate the secondary metabolites, the extracts were subjected to MS/MS-guided molecular networking analysis. Structurally diverse compounds including the polyketides 22-dehydroxymethyl-kijanolide (GH99 strain) and Abyssomicin I (GH176 strain) were detected in the extracts. Interestingly, Brevianamide F was detected in the extract of *Micromonospora*, which has previously been mostly found in fungal species. Other compounds such as cyclic tripeptides, Cyclo(l-Pro-d-Ile) and Cyclo(d-Pro-l-Phe), were also identified in this strain. In summary, for the first time, we explored the diversity of Actinobacteria and evaluated their bioactive potential from the Unkeshwar hot springs. The potent strains isolated in the study could be useful in drug discovery programs.

## Abstract 2

The bioactive potential of Actinobacteria endemic to hot springs has rarely been investigated.

## Graphical abstract

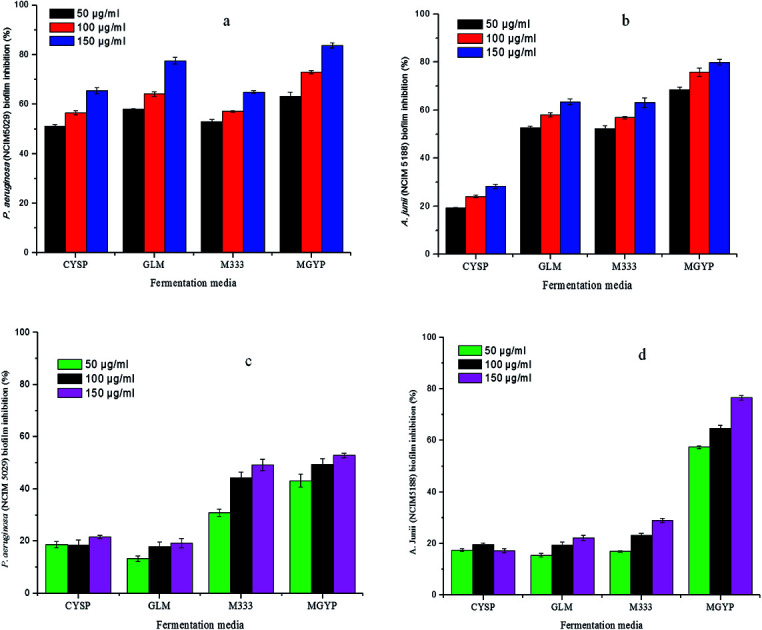

## 1 Introduction

The phylum Actinobacteria serves as a notable source of various bioactive compounds that act as therapeutic agents.^1^ Over the last few decades, due to the high rediscovery rate of known compounds, novel and alternative approaches are being pursued.^2^ Exploration of unusual and extreme environments in search of novel bioactive compounds is gaining much research attention within the scientific community.^3^ Various extreme environments and ecosystems, such as alkaline soils, hypersaline lakes, marine sponges, deep-sea sediments, mangrove ecosystems, insect intestines and medicinal plants, have been explored towards the aim of isolating biologically active metabolites.^4–6^ In this context, hot springs have rarely been studied, and very few studies have addressed the diversity and metabolic capabilities of Actinobacteria from hot spring environments.^7^ Song *et al.* (2014) showed using culture-independent studies that hot springs harbour diverse Actinobacteria with a considerable number of uncultured species.^8^ A recent culture-based study revealed that Actinobacteria associated with hot springs typically possess significant biosynthetic potential for the production of various bioactive secondary metabolites.^9^ A high microbial diversity endemic to hot spring environments implies that hot spring ecosystems should be investigated for novel microbial sources and compounds. The market demand for new drugs is extremely urgent due to the spread of antibiotic-resistant pathogens and biofilm-forming bacteria.^10^ Therefore, bioprospecting studies of hot springs may increase the possibilities of obtaining novel types of compounds with desirable activities. Exploring new microbial taxa potentially represents a successful strategy in the discovery of candidate molecules for the development of novel microbial drugs.^11^

In recent years, advanced multi-omics techniques based on next-generation sequencing, as well as novel culture-based methods, have been employed for the active exploration of microbial sources in search of secondary metabolites from extreme environments.^3,12^ A few studies have also engaged in exploring the biosynthetic potentials of novel metabolites of Actinobacteria isolated from such environments using advanced metabolomics and genome screening methods.^13^ Numerous biosynthetic gene clusters involved in the synthesis of different types of secondary metabolites such as polyketides and terpenes were identified in different species of Actinobacteria. Additionally, novel biosynthetic gene clusters have also been uncovered from known species using large-scale genomics and metabolomics studies.^14^ Along with such genome-based investigations, research groups have equally utilised sophisticated spectroscopic tools to effectively uncover the potential novelty of the active molecules. A major limitation associated with drug discovery research is repeatedly obtaining known compounds. Currently, molecular networking methods are being employed for effective chemical dereplication. Molecular networking allows for rapid comparison of mass spectrometry profiles from complex extracts and is gaining considerable attention in novel metabolite discovery research.^15^ Investigating underexplored extreme environments with a special emphasis on Actinobacteria and accessing their metabolic potentials using advanced techniques may increase the probability of finding potentially beneficial compounds for therapeutic applications.

In the present study, we explored the phylogenetic diversity of cultivable Actinobacteria isolated from the Unkeshwar hot springs, India. The isolated strains were assessed for their bioactive potential. Potent strains with strong antibacterial, anti-biofilm, and anticancer activities were further investigated for biosynthetic gene cluster and metabolite analysis utilising molecular networking tools *via* comparisons with natural products databases.

## 2 Experimental designs

### 2.1 Site description and sample collections

The Unkeshwar hot springs lie towards the South East of the Deccan Plateau; it is geographically located in central India (19°34′–19°40′N and 78°22′–78°34′E) (Fig. S1†). The temperature of the water of the hot springs was found to be in the range of 50–60 °C with pH values in the range of 6.9–7.2. Water samples were collected from the hot springs, transported to the laboratory and immediately used for the isolation of Actinobacteria.

### 2.2 Selective isolation of Actinobacteria

Isolation was carried out using different commercially available Actinobacteria-specific media, as well as other low-nutrient media (Hi-Media, India). International *Streptomyces* Project (ISP) medium (ISP1 to ISP7), starch casein agar and *Streptomyces* agar were used for isolation. Additionally, the low-nutrient media Reasoner's 2A agar (R2A) and Bushnell–Haas (BH) agar were also used. Other formulated culture media, such as nutrient agar (peptone 0.5%, beef extract 0.3%, sodium chloride (NaCl) 0.5%, agar 1.8%) and diluted Luria–Bertani (LB) broth (1 : 100 and 1 : 1000), were also used for the isolation. Water samples were pre-heated in a water bath at 50 °C for one hour and were then directly placed on the agar plates. All the plates were incubated at 37 °C and 45 °C for 7 days. After every 24 hours of incubation, plates were observed for distinct colony morphologies and distinct morphotypes of putative Actinobacteria were subcultured. Isolated colonies were repeatedly subcultured on the respective media plates to obtain the pure cultures. All isolated cultures were preserved in glycerol stock (20% v/v) at −80 °C.

### 2.3 16S rRNA gene-based identification and phylogenetic analysis

Putative Actinobacteria strains were identified using phylogenetic analysis based on 16S rRNA gene sequencing.

Briefly, genomic DNA was isolated from the pure cultures using a HiPurA™ (Hi-Media, India) DNA isolation kit following the manufacturer's protocol. PCR amplification of the 16S rRNA was performed using the universal primers 16F27 (5′CCAGAGTTTGATCMTGGCTCAG3′) and 16R1525 (5′TTCTGCAGTCTAGAAGGGGTGWTCCAGCC3′). The amplified PCR products were purified using Exosap (Affymetrix, USA) and sequenced using an ABI 3500xl genetic analyser (Invitrogen/Life Technologies). Nucleotide sequence data were analysed using BLAST with the closest sequences retrieved from the National Center for Biotechnology Information (NCBI) and EzTaxon database.^16,17^ The phylogenetic tree was constructed by a neighbour-joining method using molecular evolutionary genetic analysis (MEGA-7) software.^18^ Representative sequences were deposited in GenBank under the accession numbers listed in Table S1.†

### 2.4 Screening for antimicrobial activity

The isolated strains were screened for antimicrobial activity using a modified cross streak method^19^ against different pathogenic cultures such as *Staphylococcus epidermidis* (NCIM 2493), *Shigella flexneri* (NCIM 5265), *Klebsiella pneumoniae* (NCIM 2098), *Salmonella abony* (NCIM 2257), *Escherichia coli* (NCIM 2563), *Micrococcus luteus* (NCIM 2704), and *Candida tropicalis* (NCIM 3556). Mueller–Hinton (MH) agar plates were prepared and streaked with single Actinobacteria strains in a straight line of 2 mm width on each plate, and were incubated at 37 °C for 3 to 7 days based on the growth appearance. The plates were then streaked with the test cultures with a single streak at a 90° angle to the grown culture of Actinobacteria. The plates were then further incubated at 37 °C for 24 hours and observed for growth inhibition of the test cultures.

### 2.5 Selection of potent strains and biosynthetic potential assessment

Strains that demonstrated the highest growth inhibition of the maximum number of test cultures were selected for further study. Two strains, *Micromonospora* sp. strain GH99 and *Streptomyces* sp. strain GH176 exhibited the highest antimicrobial activity. The biosynthetic potential of these strains was analysed using PCR-based screening for polyketide synthases (PKS I & PKS II) genes. PCR amplification of the genes was performed using degenerate primer sets and PCR conditions with slight modification as described previously.^20^ PCR was performed with an initial denaturation of 95 °C for 5 min, 30 cycles of 30 s at 95 °C, 30 s at 55 °C, and 1.5 min at 72 °C, with a final extension of 10 min at 72 °C. Purification and sequencing of the amplified products was performed using the same primers as those used for PCR and using the same protocol as described for the 16S rRNA sequencing. The sequencing data were analysed by BLAST with the closest cultured sequences retrieved from the NCBI database.^16^

### 2.6 Fermentation and crude extract preparation

Crude extracts of *Micromonospora* sp. strain GH99 and *Streptomyces* sp. strain GH176 were prepared by culturing them separately in four different fermentation media. Seed cultures of these strains were prepared and 5% of these were each inoculated with 500 ml of the fermentation media. The composition of the fermentation media is provided in Table S2.† The flasks were incubated at 37 °C for 7 days in a rotary shaker incubator at 150 rpm. Fermentation broth was centrifuged at 6500*g* for 10 min and the supernatant was collected for the extraction of metabolites. Extraction was performed three times using an equal volume of ethyl acetate and the final extract was concentrated under reduced vacuum using a rotary vacuum evaporator (Heidolph, Germany). The dry residue was re-dissolved in appropriate solvents for the analysis of bioactivity and metabolites.

### 2.7 Antimicrobial activity of the extracts

The agar well diffusion method was used to determine the antimicrobial activity of the extracts against the same test cultures used in the initial screening. Mueller–Hinton (MH) agar plates with bored wells (using sterile borer) were seeded with test cultures and allowed to dry for a few minutes. In the wells bored (6 mm diameter), a 20 μl extract was added along with the controls (only ethyl acetate). The MH plates were incubated at 37 °C for 24–48 hours and were observed for the zone of inhibition around the wells bored, which was followed by measurements of the inhibition zone diameters.

### 2.8 Anti-biofilm activity of the extracts

The anti-biofilm activity of the extracts was checked against the biofilm-forming Gram-negative bacteria, *Pseudomonas aeruginosa* NCIM 5029 and *Acinetobacter junii* NCIM 5188, in a 24-well polystyrene microplate as described previously.^21^ Briefly, overnight-grown cultures were diluted to obtain 0.1 OD at 620 nm. These cultures were inoculated into the 24-well polystyrene plates containing LB media with or without different concentrations (50–150 μg ml^−1^) of the extracts. Plates were incubated in an incubator without shaking at 37 °C for 24 hours. The quantification of the biofilm inhibition was checked using crystal violet staining, scanning electron microscopy (SEM) and laser-scanning confocal microscopy (LSCM). For the quantification of adherent cells, the wells were rinsed twice with sterile distilled water, and the cells at the bottom of the wells were harvested as described earlier and were then analysed using a spectrophotometer at 570 nm. For microscopic analysis, biofilms were developed over the cover-slips kept inside the 24-well plates. After incubation, the cover-slips were washed carefully with sterile 1XPBS solution 2–3 times and then fixed overnight with 6.25% glutaraldehyde (in 50 mM phosphate buffer pH 7.4). These coverslips were then serially dehydrated in 30, 50, 80 and 100% ethanol solutions and dried sufficiently. They were then mounted on stubs and sputter coated with gold, and imaging was performed using SEM (FEI ESEM Quanta 200-3D, USA). For confocal microscopy, the biofilms that had formed over the cover slips were stained with a staining solution consisting of LIVE/DEAD® BacLight™ bacterial 109 viability kit (containing 3.34 μM SYTO 9 and 20 μM propidium iodide). LSCM imaging was performed using a 60× objective lens on an Olympus FV1000 microscope with HCS studio software of Thermo Scientific (USA). All sets of experiments were performed in triplicate and data generated were presented as the average ± standard deviation with representative images.

### 2.9 Anticancer activity of the extracts

Anticancer activity was evaluated against the breast cancer cell line MCF7. The assay was carried out as described earlier.^22^ In brief, 5000 cells were seeded per well in a 96-well plate in triplicate, and each well was treated separately with crude extracts of the strains at different concentrations (10–200 μg ml^−1^). Plates were incubated for 48 hours at 37 °C in the presence of 5% CO_2_. Twenty microlitres of MTT (3-(4,5-dimethylthiazole-2-yl)-2,5-diphenyl tetrazolium bromide) dye (5 mg ml^−1^) was added to each well and the samples were maintained for 4 hours. The reaction was then terminated by adding 200 μl of isopropanol, which dissolves the formazan crystals. Then, a reading was taken at 540 nm using an ELISA reader (Bio-Rad).

### 2.10 Metabolite analysis and molecular networking

The extracts of both strains were separately dissolved in acetonitrile (HPLC grade) and used for metabolite separation using the Accela™ ultra-high performance liquid chromatography (UHPLC) system (Thermo Fisher, Waltham, USA) using C18 Hypersil Gold column (1.9 μm, 2.1 × 150, Thermo) with a linear gradient of solvent A (acetonitrile with 0.1% of formic acid) against 2–95% of solvent B (water with 0.1% of formic acid) at a flow rate of 350 μl min^−1^ and temperature of 45 °C for 15 min. The molecular weight was identified by using a Q-Exactive-Orbitrap mass spectrometer (Thermo Fisher, Germany) in the electrospray ionisation-positive (ESI+) mode, with the mass scan range set from 100 to 1500 *m*/*z*. Data acquisition and processing were performed using Thermo Scientific Xcalibur software (Version 3.0). The tandem mass spectrometry (data-dependent MS/MS) data were collected with collision energies of between 30 and 40 eV. The raw data from the instrument were converted into the mzxml file format by using ProteoWizard (http://proteowizard.sourceforge.net/).

Molecular networking was performed using the online data analysis portal of Global Natural Products Social Molecular Networking^23^ (https://gnps.ucsd.edu). The data were filtered by removing all MS/MS peaks within ±17 Da of the precursor *m*/*z*. The MS/MS spectra were window filtered by choosing only the top 6 peaks in the ±50 Da window throughout the spectrum. The data were then clustered with MS-Cluster with a parent mass tolerance of 0.02 Da and an MS/MS fragment ion tolerance of 0.02 Da to create consensus spectra. Further, consensus spectra that contained less than 2 spectra were discarded. A network was then created, where edges were filtered to have a cosine score of above 0.7 and more than 6 matched peaks. Further edges between two nodes were retained in the network only if each of the nodes appeared in each other's respective top 10 most similar nodes. The spectra in the network were then searched against GNPS spectral libraries. The library spectra were filtered in the same manner as the input data. All matches kept between network spectra and library spectra were required to have a score above 0.7 and at least 6 matched peaks. The molecular network was visualised using Cytoscape. In parallel, de-replication of the metabolites was detected by matching spectral data with the commercial Dictionary of Natural Products (http://dnp.chemnetbase.com/) and StreptomeDB^24^ (http://www.pharmaceutical-bioinformatics.org/streptomedb).

## 3 Results and discussion

### 3.1 Selective isolation of Actinobacteria

In total, 12 different media were used for the isolation of Actinobacteria. A total of 121 isolates of putative Actinobacterial colony morphologies were selectively isolated and purified. Different types of media resulted in the isolation of various forms of colony morphologies. The pigment-producing colonies mostly appeared in the low-nutrient media after 48 hours of incubation. From all types of media, visible colonies appeared between 5 and 7 days of incubation with well-defined aerial mycelium, as well as pigmented colonies, all of which were then isolated. All of the isolated colonies were purified and underwent identification using 16S rRNA gene sequence analysis.

### 3.2 Taxonomic diversity of Actinobacteria

The 16S rRNA gene sequencing analysis revealed that out of 116 isolated strains, 86 strains were Actinobacteria, which could be grouped into 21 different genera of 15 Actinobacterial families. The overall distribution of the number of genera and strains to each family is shown in Fig. S2.† A representative strain of each genus with 16S rRNA sequence similarity, along with the type of strain and the accession number, is provided in Table S1.† The presence of taxonomically diverse members of Actinobacteria in the Unkeshwar hot springs may be due to its moderate temperature range (50–60 °C). It is well reported that thermophilic members of Actinobacteria can survive at a temperature range of 37–65 °C in such environments.^4^ Most Actinobacterial genera found in hot springs are known to form spores under adverse conditions and are hence able to survive at the high temperatures of hot springs. In our study, the maximum numbers of strains were found to belong to Streptomycetaceae (25%), Micrococcaceae (22%), Nocardioidaceae (6%) and Microbacteriaceae (5%) families (Table S3†). These results were found to be consistent with both culture-dependent and culture-independent studies of Actinobacteria from hot springs.^8,25^ The highest relative abundance (25%) of *Streptomyces* was found in this hot spring. Due to its spore forming ability, *Streptomyces* has previously been found to be a dominant member in various extreme habitats, including the intertidal zones of marine ecosystems and arid soils.^26,27^ Apart from *Streptomyces*, the most considerable number of distinct genera was obtained for Micrococcaceae and Nocardioidaceae families. *Arthrobacter*, *Micrococcus*, *Microbacterium* and *Nocardioides* genera were represented with a relatively higher number of strains. Notably, strains belonging to a few rare genera such as *Actinomadura*, *Cellulomonas*, *Cellulosimicrobium*, *Janibacter*, *Rothia*, *Barrentisimonas*, *Dietzia*, and *Glycomyces* were also recovered in this study (Fig. S2b†). A few genera such as *Micromonospora* and *Glycomyces* have rarely been reported in hot spring environments. The genus *Micromonospora* is a well-known antibiotics producer and has been found to be a common inhabitant of aquatic environments, with various species producing novel bioactive compounds.^28^ The *Glycomyces* species has been reported from the intertidal zones of marine ecosystems.^26^ Although a few studies have also reported presence of *Glycomyces* species in hot spring environments, none of these have reported their isolation from hot springs.^6,7^ Phylogenetic relationships of the representative strains of each genus were assessed using neighbour-joining (NJ) methods (Fig. S3†). All the identified strains showed 16S rRNA gene sequence similarities in the range of 97.6–100% with the sequences present in the curated database (Table S1†). The strains of *Streptomyces* shared around 98.6–100% sequence similarity, whereas non-*Streptomyces* members shared 98.6–100% similarity with the known strains. Some of the strains of *Aeromicrobium*, *Dietzia*, *Microbacterium* and *Arthrobacter* showed 98–99% similarities with the strains in the database. One strain (UAC28) displayed a 97.6% similarity with the *Marmoricola* species.

### 3.3 Screening for antimicrobial activity

The antimicrobial activity results were recorded based on the growth inhibition of the test cultures. In total, 28 strains including *Streptomyces* sp. (21 strains), *Micromonospora* sp. (2 strains), *Rhodococcus* sp. (2 strains) and one strain of *Aeromicrobium* sp. showed antimicrobial activity against at least one of the test cultures (Fig. S4a†). Among the 21 positive *Streptomyces* strains, 3 strains inhibited the growth of all the test cultures. However, two strains (*Micromonospora* sp. strain GH99 and *Streptomyces* sp. strain GH176) exhibited the highest activity. In general, both of these genera (*Streptomyces* and *Micromonospora*) are well known to produce a wide range of secondary metabolites. Most reported antibiotics have been isolated from the *Streptomyces* species.^29^ However, most of these antibiotic-producing strains have been isolated either from soil or freshwater ecosystems. Until now, only a few *Streptomyces* species have been reported from hot springs. A thermo-tolerant *Streptomyces* sp. Al-Dhabi-1 producing bioactive compounds and showing growth inhibition of *S. agalactiae* and *K. pneumoniae* was reported from the Tharban hot spring.^30^ Recently, a few thermo-tolerant *Streptomyces* species, such as *S. tauricus*, *S. toxytricini*, *S. coeruleorubidis* and *S. lanatus*, with high antimicrobial potentials have also been isolated from high temperature environments such as desert soils. Additionally, it has also been documented that desert soils host several other thermo-tolerant members of Actinobacteria such as *Micromonospora*, *Actinomadura* and *Streptosporangium*, which could also act as a potential source of bioactive compounds.^31^ In our study, we also found *Micromonospora* sp. as well other genera with antimicrobial potentials, indicating that thermal aquatic ecosystems could also serve as a potential source of antibiotic-producing microorganisms.

### 3.4 Selection of potent strains and biosynthetic potential assessment

During the antimicrobial screening, *Micromonospora* sp. strain GH99 and *Streptomyces* sp. strain GH176 exhibited the highest antimicrobial activity. These strains were further evaluated for PCR-based biosynthetic potential for PKS I and PKS II gene analysis. Both strains were found to be positive for PKS I genes (Fig. S4b†). A sequence similarity search of the PKS I gene showed that the strain GH176 shared 94–96% similarity with sequences retrieved from the database. Interestingly, the *Micromonospora* strain found to have <79% similarity with the retrieved sequences from the NCBI database, indicating its potential novelty. Phylogenetic analysis revealed that *Streptomyces* sp. and *Micromonospora* sp. strains clustered with the respective PKS-I genes of known strains of *Streptomyces* sp. and *Micromonospora* sp. in the NCBI database (Fig. S5†). Examining the biosynthetic genes involved in the production of the bioactive compounds has extensively been studied in different *Streptomyces* species.^32^ In fact, the genome of *Streptomyces* has been found to possess multiple copies of PKS and NRPS genes. However, the functional roles of some of these clusters have still not been explored. The species of *Micromonospora* were also reported with PKS domains in their genomes, but they have been studied rarely when compared to the *Streptomyces* species.^33^ In the present study, both strains were found to be positive for the PKS biosynthetic cluster and also exhibited antimicrobial activity. However, the high dissimilarity in the PKS I gene sequence of both the strains indicates that these strains might possibly have a high level of sequence divergence. Alternatively, these strains may also possess other biosynthetic pathways involved in the synthesis of secondary metabolites. It is worth noting that other biosynthetic gene clusters are also found to be present in different species of Actinobacteria, and might be involved in the synthesis of antimicrobial compounds. Nevertheless, only limited numbers of PKS genes corresponding to known bioactive compounds have been identified so far from hot spring environments.^8^

### 3.5 Crude extract preparation and bioactivities

**Fig. 1:**
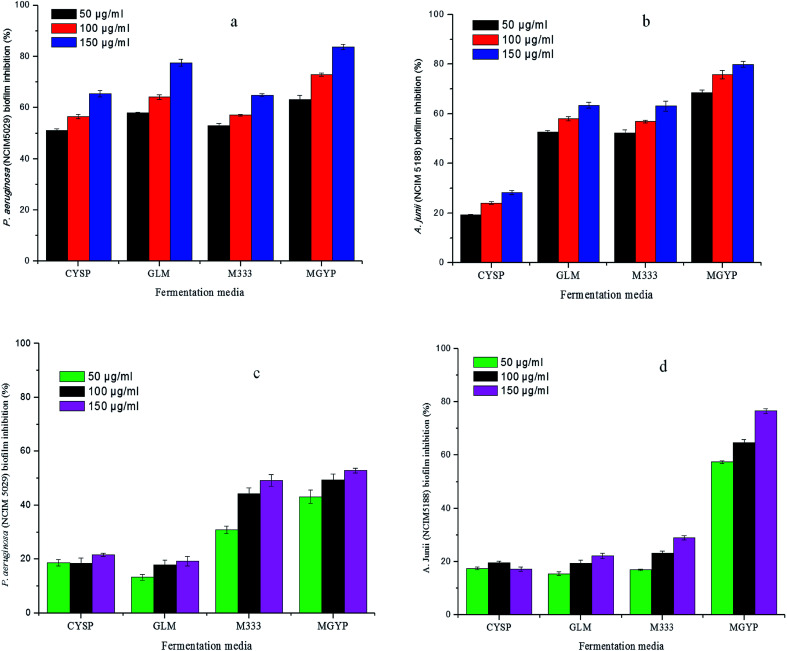
Biofilm inhibition (%) of *P. aeruginosa* NCIM 5029 and *A. junii* NCIM 5188 by extract of GH99 strain (a and b) and GH176 strain (c and d) prepared using different fermentation media at different concentrations.

**Fig. 2:**
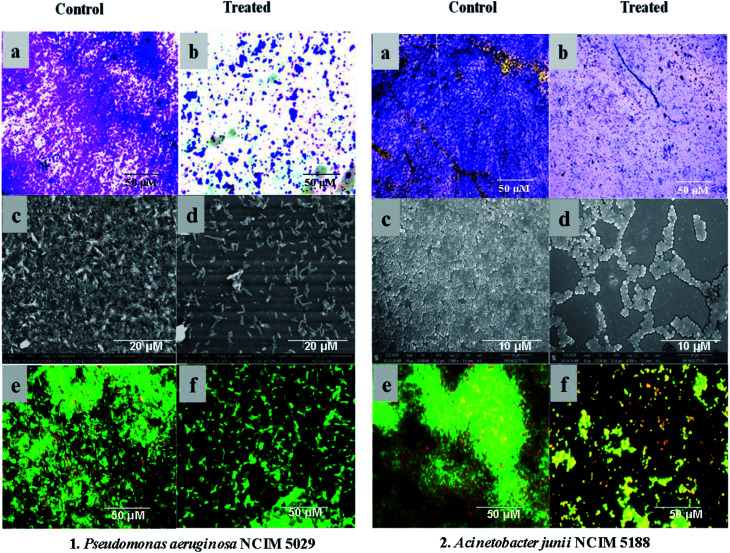
Visualization of the biofilm inhibition: (1) *P. aeruginosa* NCIM 5029, and (2) *A. junii* NCIM 5188. Images (a) and (b) represent the crystal violet staining of the biofilms in the control and treated samples, respectively. Images (c) and (d) display the SEM images of the control and treated samples, respectively. Images (e) and (f) are the laser scanning confocal microscopic views of the control and treated samples, respectively, showing live cells as green and dead cells as red with biofilm inhibition in the treated samples.

Crude extracts of both potent strains, *Streptomyces* sp. GH176 and *Micromonospora* sp. GH99, were prepared using different fermentation media. The extract obtained from MGYP medium (strain GH176) and Medium 333 (strain GH99) were found to be more effective for antimicrobial activity against *S. epidermis* NCIM 2493 (Fig. S4c†) as well as other pathogenic bacteria (Table S4†). At lower concentrations (50–150 μg ml^−1^) MGYP medium extracts exhibited the highest biofilm inhibition of *P. aeruginosa* NCIM 5029 and *A. junii* NCIM 5188. A notable reduction in biofilm formation was observed after 24 hours of incubation. The extracts of the *Streptomyces* sp. GH176 strain showed 52.9 ± 0.8% and 76.5 ± 0.8% biofilm inhibition of *P. aeruginosa* NCIM 5029 and *A. junii* NCIM 5188, respectively (Fig. 1). The extract of *Micromonospora* exhibited the highest reduction in the biofilm (83.65 ± 1.04%) of *P. aeruginosa* NCIM 5029 at 150 μg ml^−1^ concentration of the extract (Fig. 1). The crystal violet staining results also clearly demonstrated the inhibition of biofilm formation (Fig. 2a and b). Further, we precisely checked biofilm inhibition by employing microscopic techniques. Using SEM analysis, we observed the impact structure of the biofilm matrix in the untreated samples. However, in the treated samples, the matrix-like structure of the biofilm was significantly reduced. In SEM images of the treated samples, the cells were found to be more dispersed as compared to the compact structure of the biofilms in the untreated samples (Fig. 2c and d). To further confirm the results of the reduction in the formation of the biofilms, we performed confocal microscopy studies using propidium iodide and SYTO green staining to differentiate the live and dead cells. In this case, the control samples (untreated) also showed the formation of a condensed biofilm. In the treated samples, more dispersed live cells (stained green) were observed and only a few numbers of cells were found to be dead (stained red) (Fig. 2e and f). The results indicate that the extracts interfered with the biofilm formation of *P. aeruginosa* NCIM 5029 and *A. junii* NCIM 5188 test cultures. A few studies have demonstrated that *Streptomyces* species modulate biofilm formation in *P. aeruginosa* and *S. aureus* cultures.^34,35^*Micromonospora* species have rarely been reported for their anti-biofilm activities; however, our strain showed higher biofilm inhibition as compared to the *Streptomyces* strain. We also tested the anticancer activity of the extracts using the breast cancer cell line MCF7. The results demonstrated that the extracts were also found to be effective against the cancer cells. Around 50% cell death was observed for both extracts at a concentration of 100 μg ml^−1^ within 48 hours. The extract of *Micromonospora* exhibited marginally higher activity as compared to the *Streptomyces* strain (Fig. S6†). It could therefore be inferred that *Micromonospora* species from unusual habitats could provide greater opportunities for the discovery of novel microbial compounds.

### 3.6 Molecular networking and metabolite analysis

**Fig. 3:**
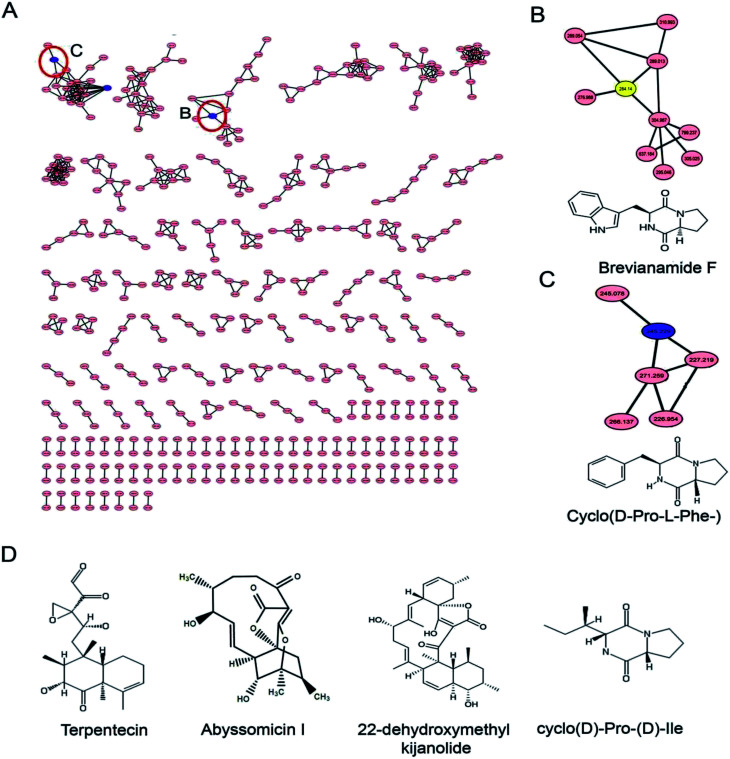
Metabolite analysis of the extracts: (A) molecular network of the extracts of *Micromonospora* sp. GH99 and *Streptomyces* sp. GH176 strains; (B) and (C) show the enlarged sub-network view of the compounds Brevianamide F and Cyclo(d-Pro-l-Phe-) in the cluster; (D) chemical structure of compounds dereplicated using GNPS, StreptomeDB and DNP databases.

**Fig. 4:**
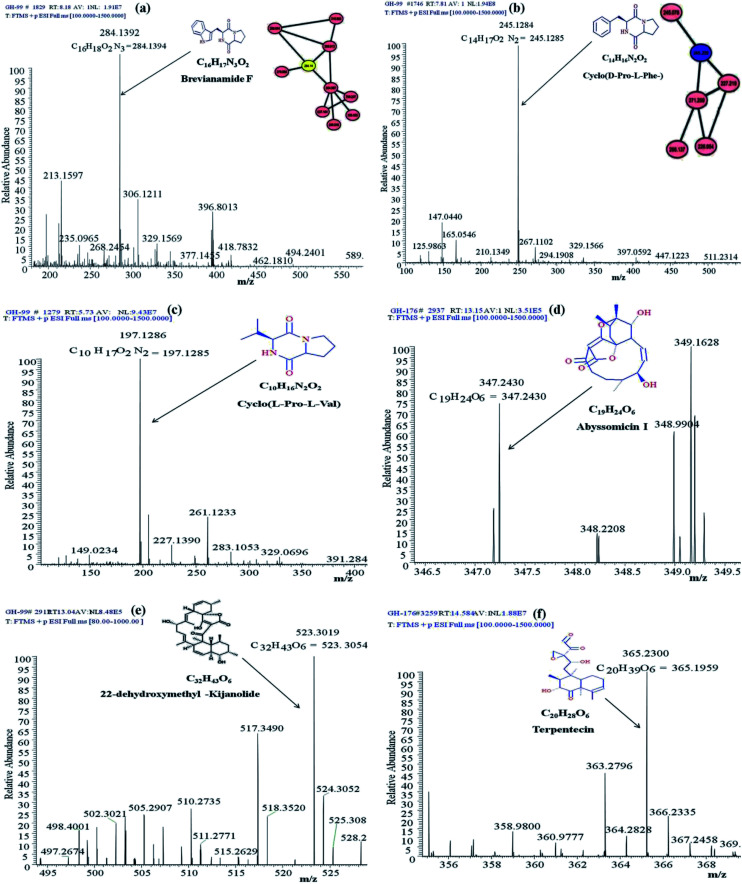
Annotated MS/MS spectra acquired by UHPLC-Orbitrap MS in positive mode of (a) Brevianamide F (*m*/*z* [M + H]^+^ 284.1392), (b) Cyclo(d-Pro-l-Phe-) (*m*/*z* [M + H]^+^ 284.1392), (c) Cyclo(l-Pro-l-Val-) (*m*/*z* [M + H]^+^ 197.1286), (d) Abyssomicin I (*m*/*z* [M + H]^+^ 347.2430), (e) 22-dehydroxymethyl-kijanolide (*m*/*z* [M + H]^+^ 523.3019), and (f) Terpentecin (*m*/*z* [M + H]^+^ 365.23).

Identification of the bioactive metabolites in the extracts was performed using ultra-high performance liquid chromatography (UHPLC) along with molecular weight determination in the electrospray ionisation-positive (ESI+) mode (Fig. S7†). The dereplication of the chemical profiles of both extracts was done by analysing the HRMS data using molecular networking and comparing with the commercial Dictionary of Natural Products and StreptomeDB databases (Fig. 3). The analysis led to the identification of several known and unknown metabolites in the extracts of both strains. In total, seven different compounds with high peak intensities were detected. The identities of these compounds were confirmed by comparing the accurate mass acquired by UHPLC-Orbitrap MS and using a dereplication strategy (Fig. 4 and Table 1). The MS/MS spectra of the compounds were matched and confirmed with the available database (Table S5†). For most other compounds, no relative match was found in the database, possibly indicating that highly diverse kinds of metabolites are produced by these strains. The identified compounds from the extract of *Streptomyces* sp. strain GH176 were found to belong to different natural product categories, including polyketides (Abyssomicin), terpenes and terpenoids (Terpentecin). Previous studies have also shown the detection of these classes of compounds, such as Abyssomicin I, a modified polycyclic polyketide, and Terpentecin, an inhibitor of DNA synthesis in different *Streptomyces* species.^36,37^ In *Micromonospora*, polyketides such as 22-dehydroxymethyl-kijanolide, as well other compounds of pharmaceutical interest including Brevianamide F and cyclic tripeptides (Cyclo(l-Pro-d-Ile) and Cyclo(d-Pro-l-Phe)), were also identified (Table 1). Interestingly, Brevianamide F was detected and reported as a cardio-protective compound as well as an antibacterial agent; however, previously it has mostly been isolated from fungal species.^38^ Here, for the first time, we detected Brevianamide F in *Micromonospora* species. Another compound catalysed by the PKS-I module, 22-dehydroxymethyl-kijanolide, which is a type of spirotetronate aglycone, was also detected in the extract of *Micromonospora*. However, until now, only a few previous studies have reported the isolation of 22-dehydroxymethyl-kijanolide from the *Micromonospora* species.^39^ The genus *Micromonospora* is a well-known producer of diverse classes of antibiotics such as aminoglycosides, macrolides and ansamycins.^40^ A few recent studies have shown that the species of *Micromonospora* isolated from various extreme environments produces bioactive metabolites, which may be potential candidate antibiotics, such as butremycin and the protonated aromatic tautomer of 5′-methylthioinosine (MTI).^41^ It therefore could be inferred that *Micromonospora* species from extreme environments are involved in the synthesis of a variety of novel secondary metabolites, which could act as potential candidate drug molecules. Additionally, a few small molecules such as cyclic peptides were also detected in the crude extract of the *Micromonospora* strain. Cyclic peptides are of significant importance due to their medical properties, especially those containing isodityrosine, as they have diverse biological activities, including antimicrobial, antiviral and antitumor activities.^42^ However, these compounds have rarely been isolated from Actinobacteria. In our study, the extract of *Micromonospora* sp. exhibited various bioactivities and various metabolites were detected, which demonstrates the potential of such strains for producing bioactive compounds. Overall, for the first time, we explored the diversity of Actinobacteria and evaluated their bioactive potential from this hot spring. The potent strain isolated in the study could be useful in drug discovery programs.

**Table 1:** Compounds identified after dereplication using GNPS, StreptomeDB and DNP databases along with the reported biological activities

Actinobacterial strains	Name of the compound	[M + H]^+^	Reported biological activity
*Micromonospora* sp. GH99	Brevianamide F	284.14	Cardio-protective, antibacterial activity^38^
	22-Dehydroxymethyl-kijanolide	523.30	Antitumor activity^39^
	Cyclo(l-Pro-l-Val)	197.13	Antifungal, antibacterial activity^42^
	Cyclo(d-Pro-l-Phe)	245.22	Antibacterial activity^43^
*Streptomyces* sp. GH176	Abyssomicin I	347.36	Antitumor activity^36^
	Terpentecin	365.43	Antibacterial activity^37^

## 4 Conclusions

Microbiological exploration of extreme and unusual environments for bioactive potential is an important approach for finding novel compounds. Here, we showed that the Unkeshwar hot springs host a diverse range of Actinobacteria, predominantly Streptomycetaceae, Micrococcaceae and Nocardioidaceae family members. The presence of such diverse Actinobacteria possibly suggests that hot springs could represent a valuable microbial resource for drug discovery. Two potent strains, *Streptomyces* sp. GH176 and *Micromonospora* sp. GH99, demonstrated the presence of biosynthetic genes for the production of bioactive compounds. These strains also exhibited antimicrobial properties, anti-biofilm activity against pathogenic bacteria and anticancer activity against the MCF7 cell line. Metabolite analysis using an effective dereplication strategy revealed the presence of various compounds in the extracts of both strains. Compounds such as polyketides and small cyclopeptides were detected, which suggests the bioactive potential of these strains. We conclude that the Unkeshwar hot springs host a diverse range of Actinobacteria, and some of them were found to have potential for drug discovery programs.

## Conflicts of interest

The authors declare no conflicts of interest.

## Supplementary Material

RA-009-C8RA09449G-s001
