# Supplementary material for: Bioactivities and molecular networking-based elucidation of metabolites of potent actinobacterial strains isolated from the Unkeshwar geothermal springs in India
Source: RSC Adv. 2019 Mar 28;9(17):9850–9. doi: 10.1039/c8ra09449g (PMC9062624; doi:10.1039/c8ra09449g)
Supplement: RA-009-C8RA09449G-s001 [file RA-009-C8RA09449G-s001.pdf]

Electronic Supplementary Material (ESI) for RSC Advances.
This journal is © The Royal Society of Chemistry 2019

Electronic Supplementary Material (ESI) for RSC Advances.
This journal is © The Royal Society of Chemistry 2018

Diversity, bioactivities and molecular networking based elucidation of metabolites of the potent actinobacterial strains isolated from Unkeshwar geothermal spring, India.

Gajanan T. Mehetre[a,b], Vinodh J S[a], Bhushan Burkul[c], Desai D[d], Santhakumari B[c], Mahesh S. Dharne[a,b], Syed G. Dastager[a,b*]

[a] NCIM Resource centre, CSIR-National Chemical Laboratory, Pune, India

[b] Academy of Scientific and Innovative Research (AcSIR), New Delhi, India

[c] Centre for Materials Characterization, CSIR-National Chemical Laboratory, Pune, India

[d] National center for nanoscience and nanotechnology, University of Mumbai, India

Supplementary Tables

Table S1 The 16S rRNA gene sequence similarity with validly published type strains (EzTaxon database) of the representative strains of each genus along with the accession numbers.

Sr. No	Strain name	Closest match in the database (EzTaxon)	16S rRNA gene Similarity (%)	Accession numbers
1	GHP2B	*Brevibacterium pityocampae* DSM 21720 (T) (EU484189)	99.56% (1357/1363)	MF101689
2	GHP77	*Cellulomonas carbonis* T26(T) (HQ702749)	99.54% (1309/1315)	MF101705
3	UAC259	*Corynebacterium coyleae* DSM 44184(T)(X96497)	100.00% (706/706)	KT345659
4	U58	*Barrientosiimonas humi* 39(T)(JF346171)	99.93% (1438/1439)	KT345635
5	GH81	*Dietzia schimae* YIM 65001(T) (EU375845)	98.64% (1380/1399)	MF101695
6	GM17	*Glycomyces lechevalierae* NRRL B-16149(T) (AY462041)	99.21% (1373/1384)	MH236182
7	GHP2	*Janibacter indicus* 0704P10-1(T) (HM222655)	99.78% (1333/1336)	MF101680
8	UAC17	*Microbacterium invictum* DC-200(T)(AM949677)	98.79% (1393/1410)	KT345651
9	GH12	*Arthrobacter globiformis* NBRC 12137(T) (BAEG01000072)	98.60% (1334/1353)	MF101713
10	U67	*Kocuria palustris* DSM 11925(T) (Y16263)	100.00% (998/998)	KT345636
11	GP2	*Micrococcus terreus* CGMCC 1.7054(T) (jgi.1058018)	99.52% (1437/1444)	MF101699
12	GHP3	*Rothia dentocariosa* ATCC 17931(T)(CP002280)	99.92% (1313/1314)	MF101707
13	GH99	*Micromonospora aurantiaca* ATCC 27029(T) (CP002162)	99.65% (1432/1437)	MF101683
14	UAC16	*Gordonia terrae* NBRC 100016(T) (BAFD01000032)	99.78% (1423/1426)	KT345650
15	UAC255	*Rhodococcus yunnanensis* YIM 70056(T) (AY602219)	99.84% (638/639)	MH236179
16	GH118	*Aeromicrobium ponti* HSW-1(T) (AM778683)	98.85% (1379/1395)	MF101688
17	UAC28	*Marmoricola pocheonensis* Gsoil 818 (T) (GQ339906)	97.64% (1327/1359)	MH236180
18	UAC 5	*Nocardioides aromaticivorans* H-1 (T) (AB087721)	99.70% (949/951)	MH236181
19	GH119	*Cellulosimicrobium funkei* ATCC BAA-886 (T) (AY501364)	99.31% (734/739)	MF101704
20	GH176	*Streptomyces gancidicus* NBRC15412(T) (AB184660)	99.51% (1427/1434)	MH236178
21	GH71	*Actinomadura apis* IM17-1(T) (AB557596)	99.68% (622/624)	MF101706

Table S2 Composition of the fermentation media used for the extract preparation

Fermentation media	Composition (gm/L)
**GLM medium**	Yeast extract: 3, malt extract: 3, peptone: 5, glucose: 10 (pH- 7.2)
**CYSP medium**	Casein hydrolysate:10, starch: 10, yeast extract: 1, peptone: 1, (pH- 7.2)
**MGYP medium**	Maltose : 3, glucose: 10gm, yeast extract: 3, peptone: 5, (pH- 7.2)
**Medium 333**	Glucose: 5, peptone: 3, soluble starch: 10, yeast extract 3, $CaCO_3$: 2, NH_4NO_3: 3, (pH- 7.2)

Table S3 Distribution of the number of genera and species to each family along with the relative abundance

Sr. No	Actinobacterial family	Actinobacterial genus	Number of species	Relative abundance (%)
1	*Brevibacteriaceae*	*Brevibacterium*	1	1.20
2	*Cellulomonadaceae*	*Cellulomonas*	1	1.20
3	*Corynebacteriaceae*	*Corynebacterium*	1	1.20
4	*Dermacoccaceae*	*Barrientosiimonas*	1	1.20
5	*Dietziaceae*	*Dietzia*	3	3.60
6	*Glycomycetaceae*	*Glycomyces*	1	1.20
7	*Intrasporangiaceae*	*Janibacter*	1	1.20
8	*Microbacteriaceae*	*Microbacterium*	5	6.00
9	*Micrococcaceae*	*Arthrobacter*	3	17.07
		Kocuria	1	
		Micrococcus	4	
		Rothia	1	
10	*Micromonosporaceae*	*Micromonospora*	3	8.50
11	*Nocardiaceae*	*Gordonia*	1	6.00
		Rhodococcus	3	
12	*Nocardioidaceae*	*Aeromicrobium*	1	17.07
		Mormoricola	1	
		Nocardioides	2	
13	*Promicromonosporaceae*	*Cellulosimicrobium*	1	1.20
14	*Streptomycetaceae*	*Streptomyces*	15	25.60
15	*Thermomonosporaceae*	*Actinomadura*	1	1.20

Table S4 Antimicrobial activity of the extracts showing the growth inhibition of test cultures based on the diameter of the zone of inhibition

Actinobacteria Strain	Media*	Test organisms# (zone of inhibition in (mm)						
		**1**	**2**	**3**	**4**	**5**	**6**	**7**
Micronomospora sp. GH99	GLM	13	19	14	15	18	13	ND
	MGYP	12	24	16	15	21	22	ND
	M333	18	21	19	22	21	23	ND
	CYSP	16	12	14	15	20	21	ND
Streptomyces sp. GH176)	GLM	10	12	13	16	12	17	13
	MGYP	23	21	18	16	19	23	18
	M333	18	22	12	ND	ND	18	17
	CYSP	ND	19	19	12	18	10	10

Foot note:

(*): Production media used for fermentation and extraction, GLM: Glucose-yeast extract-malt medium, MGYP: Malt extract-Glucose-Yeast extract-Peptone medium, CYSP: Casein hydrolysate-Yeast extract-Starch-Peptone. M333: Wort Medium (HiMedia), ND: not detected

(#): Test microorganism used for antimicrobial activity, **1:** *Escherichia coli* (NCIM2563), **2:** *Staphylococcus epidermidis* (NCIM2493), **3:** *Shigella flexneri* (NCIM5265), **4:** *Klebsiella pneumonia* (NCIM2098), **5:** *Salmonella abony* (NCIM2257), **6:** *Micrococcus luteus* (NCIM2704), **7:** *Candida tropicalis* (NCIM3556).

Table S5 MS/MS spectra of the compounds matched with the online database and Mass Frontier

Sr. No.	Metabolites	Mol. formula	MS-MS fragments match	Reference
1	Brevianamide F	$C_{16}H_{17}N_3O_2$	284.13, 267.11, 170.06, 130.06, 118.06, 98.06	GNPS Database
2	cyclo(D-Pro-L-Phe	$C_{14}H_{16}N_2O_2$	245.12,217.13,154.07, 120.08,91.05	GNPS Database
3	cyclo(L-Pro-L-Val)	$C_{10}H_{16}N_2O_2$	197.12,169.13,154.07, 124.11, 113.07,84.08	GNPS Database
4	22-dehydroxymethyl - Kijanolide	$C_{32}H_{42}O_6$	523.30,523.30,385.19, 349.16, 111.08, 85.06	Mass Frontier
5	Abyssomicin I	$C_{19}H_{24}O_6$	347.24,305.08,275.07,219.01,203.05, 185.04,152.03	Mass Frontier
6	Terpentecin	$C_{20}H_{28}O_6$	365.23,319.12, 251.09, 171.07,141.09,71.09	Mass Frontier

Supplementary figures

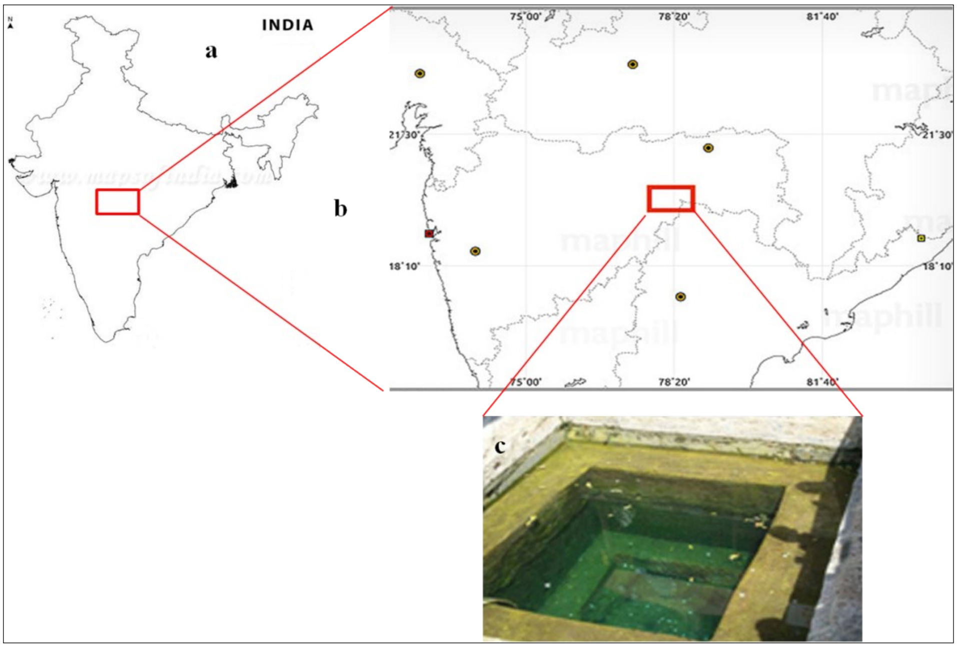

Fig.S1 Description of the sampling site a: a map of India showing the location of hot spring, b: highlights the Unkeshwar region in the map, c: Unkeshwar hot spring

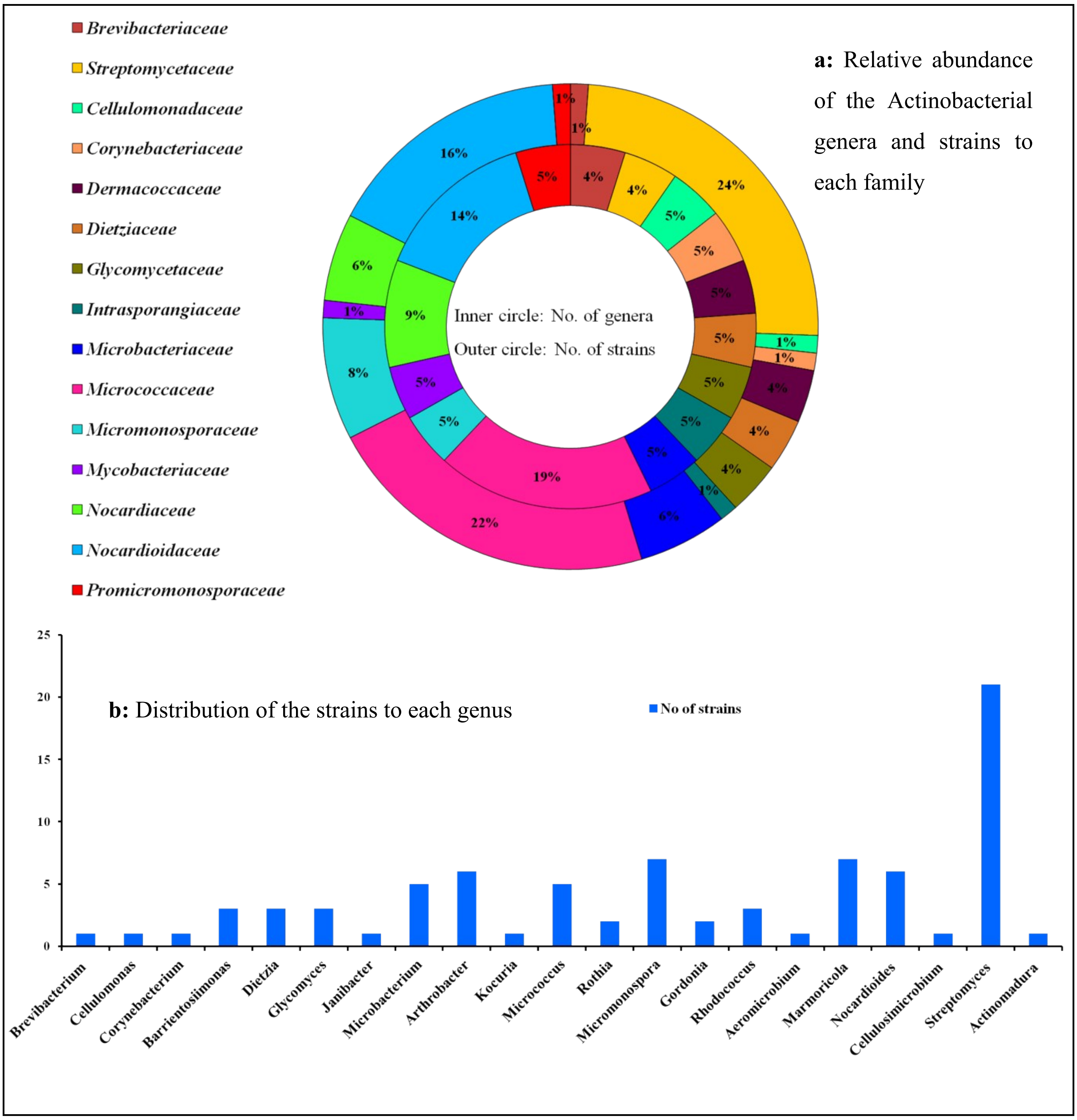

Fig. S2 Distribution of the number of genera and strain to each actinobacterial family (a), Number of strains distributed to each actinobacterial genus (b)

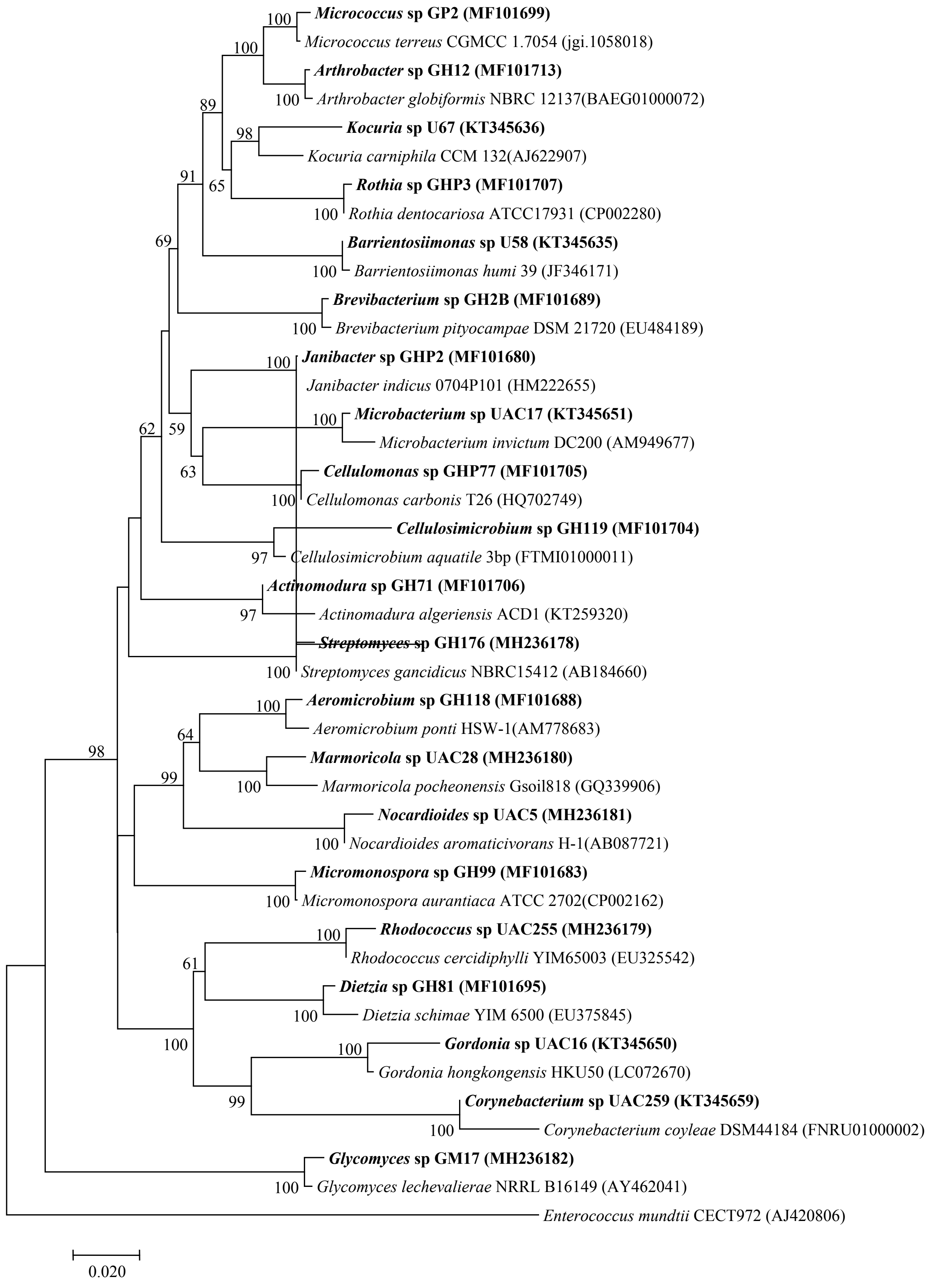

Fig. S3 Neighbor-joining phylogenetic tree based on 16S rRNA gene sequences of the representative strains with bootstrap values expressed as percentages of 1000 replications

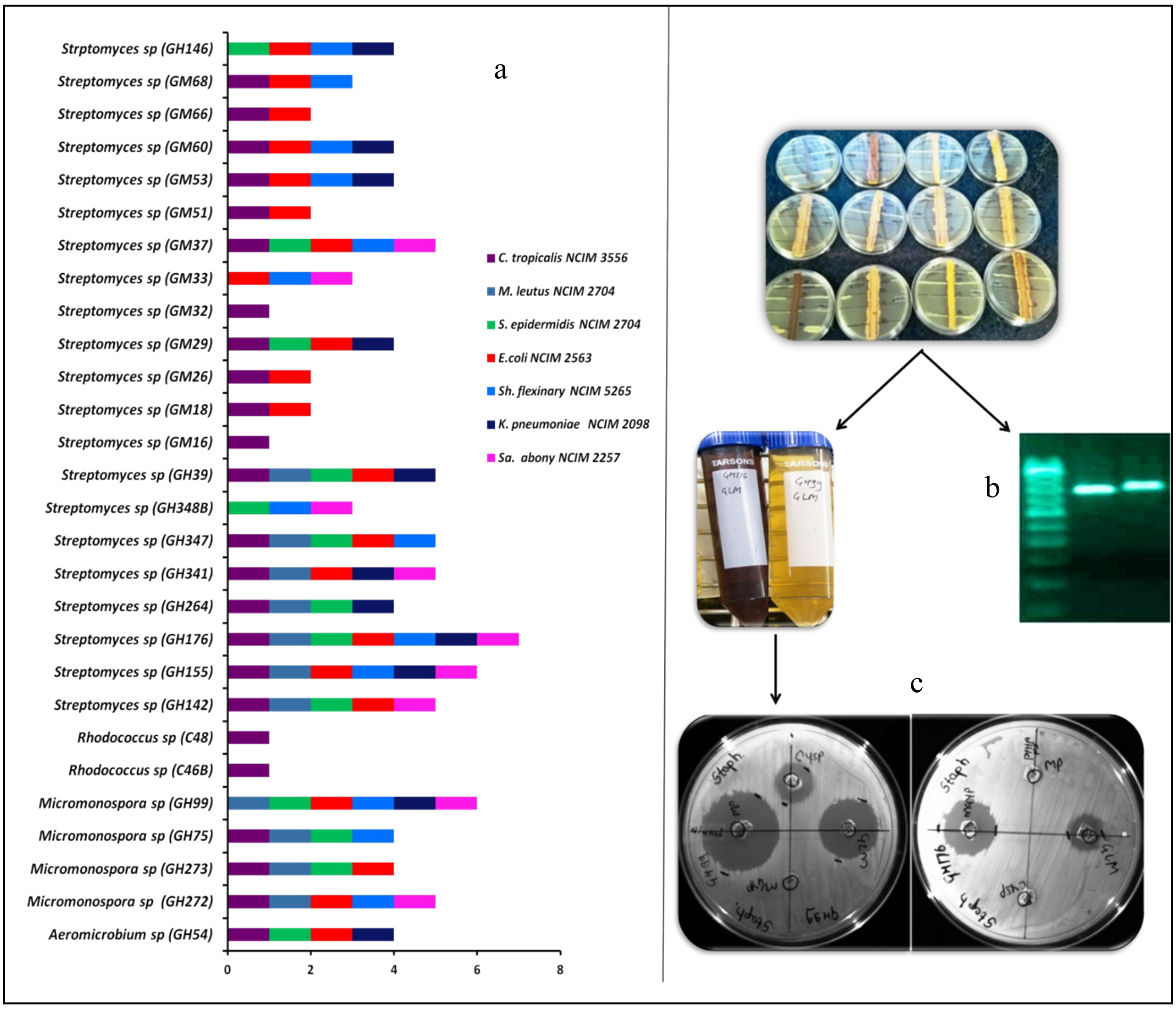

Fig. S4 Antimicrobial screening of the isolated strains, a: number of positive strain showing antimicrobial activity against the pathogenic cultures, b: a gel image of PKS-I gene of two potent GH99 and GH176 strains, c: Showing the antimicrobial activity of the extract against staphylococcus epidermis of the extract prepared from GH99 and GH176 strains

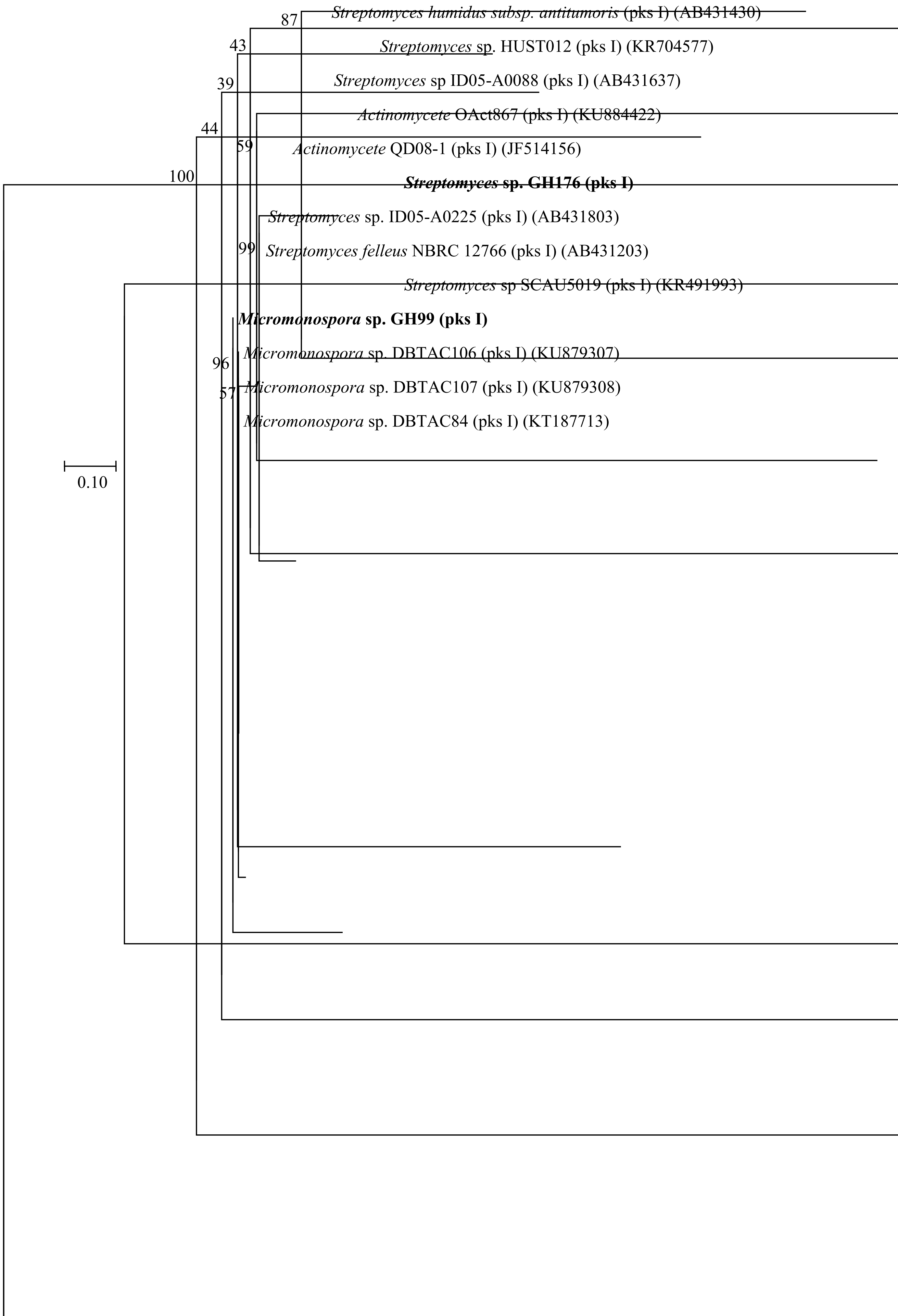

Streptomyces humidus subsp. antitumoris (pks I) (AB431430)
Streptomyces sp. HUST012 (pks I) (KR704577)
Streptomyces sp ID05-A0088 (pks I) (AB431637)
Actinomycete OAct867 (pks I) (KU884422)
Actinomycete QD08-1 (pks I) (JF514156)
***Streptomyces* sp. GH176 (pks I)**
Streptomyces sp. ID05-A0225 (pks I) (AB431803)
Streptomyces felleus NBRC 12766 (pks I) (AB431203)
Streptomyces sp SCAU5019 (pks I) (KR491993)
***Micromonospora* sp. GH99 (pks I)**
Micromonospora sp. DBTAC106 (pks I) (KU879307)
Micromonospora sp. DBTAC107 (pks I) (KU879308)
Micromonospora sp. DBTAC84 (pks I) (KT187713)
87
43
39
44
59
100
99
96
57
0.10

Fig. S5 Neighbor-joining phylogenetic tree of the PKS gene (PKS-I) showing the cluster of closest neighbor of the database. Bootstrap values were expressed as percentages of 1000 replications

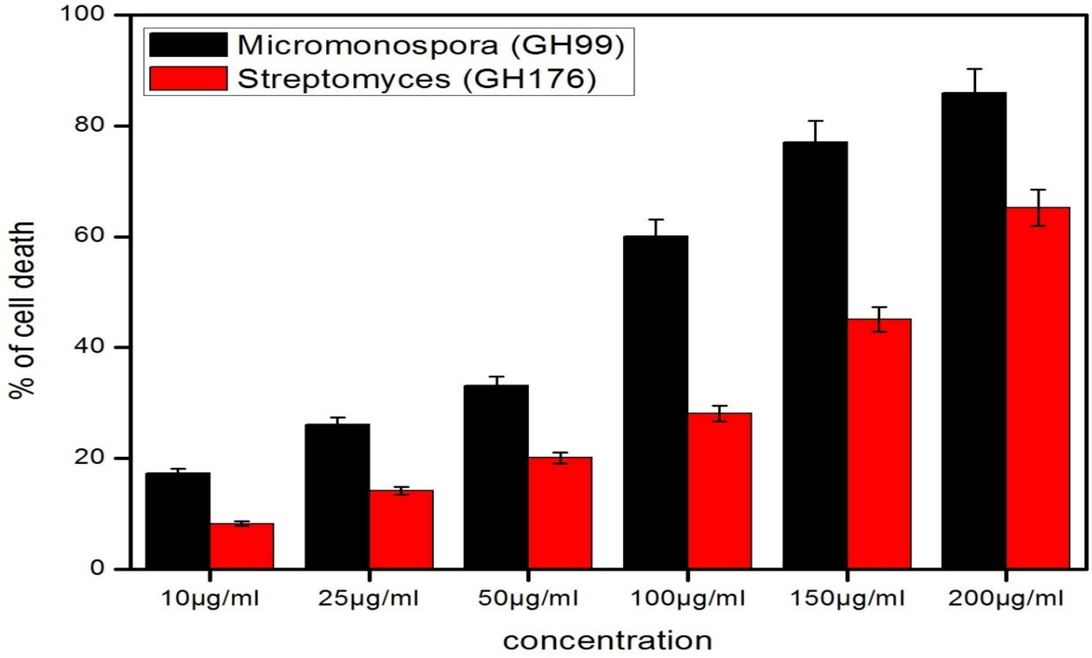

Fig. S6 Screening for anticancer potential of the extracts of *Micronomospora* sp. strain GH99 (black) and *Streptomyces* sp. GH176 (red) strains against breast cancer cell line MCF7

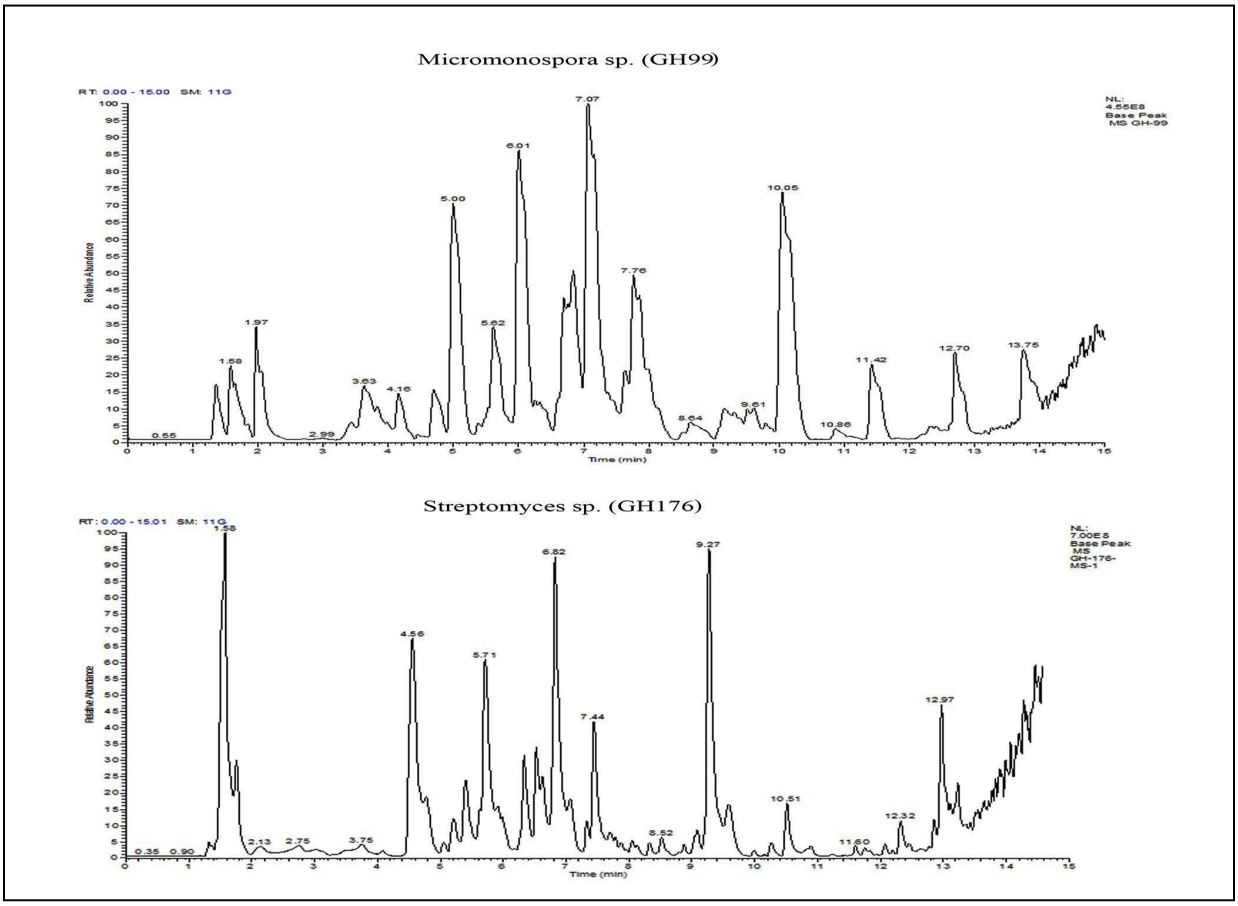

Fig. S7 LC-MS profiling of the crude extracts obtained from the *Micromonospora* sp. GH99 and *Streptomyces* sp. GH176 strains
